# Support Vector Machine Based Monitoring of Cardio-Cerebrovascular Reserve during Simulated Hemorrhage

**DOI:** 10.3389/fphys.2017.01057

**Published:** 2018-01-05

**Authors:** Björn J. P. van der Ster, Frank C. Bennis, Tammo Delhaas, Berend E. Westerhof, Wim J. Stok, Johannes J. van Lieshout

**Affiliations:** ^1^Department of Internal Medicine, Academic Medical Center, University of Amsterdam, Amsterdam, Netherlands; ^2^Department of Medical Biology, Academic Medical Center, University of Amsterdam, Amsterdam, Netherlands; ^3^Laboratory for Clinical Cardiovascular Physiology, Center for Heart Failure Research, Academic Medical Center, Amsterdam, Netherlands; ^4^Department of Biomedical Engineering, Maastricht University, Maastricht, Netherlands; ^5^MHeNS School for Mental Health and Neuroscience, Maastricht University, Maastricht, Netherlands; ^6^CARIM School for Cardiovascular Diseases, Maastricht University, Maastricht, Netherlands; ^7^Department of Pulmonary Diseases, Institute for Cardiovascular Research, ICaR-VU, VU University Medical Center, Amsterdam, Netherlands; ^8^MRC/Arthritis Research UK Centre for Musculoskeletal Ageing Research, School of Life Sciences, The Medical School, University of Nottingham Medical School, Queen's Medical Centre, Nottingham, United Kingdom

**Keywords:** cardiovascular modeling, cerebrovascular, hypovolemia, lower body negative pressure, machine learning, support vector machine

## Abstract

**Introduction:** In the initial phase of hypovolemic shock, mean blood pressure (BP) is maintained by sympathetically mediated vasoconstriction rendering BP monitoring insensitive to detect blood loss early. Late detection can result in reduced tissue oxygenation and eventually cellular death. We hypothesized that a machine learning algorithm that interprets currently used and new hemodynamic parameters could facilitate in the detection of impending hypovolemic shock.

**Method:** In 42 (27 female) young [mean (sd): 24 (4) years], healthy subjects central blood volume (CBV) was progressively reduced by application of −50 mmHg lower body negative pressure until the onset of pre-syncope. A support vector machine was trained to classify samples into normovolemia (class 0), initial phase of CBV reduction (class 1) or advanced CBV reduction (class 2). Nine models making use of different features were computed to compare sensitivity and specificity of different non-invasive hemodynamic derived signals. *Model features included*: volumetric hemodynamic parameters (stroke volume and cardiac output), BP curve dynamics, near-infrared spectroscopy determined cortical brain oxygenation, end-tidal carbon dioxide pressure, thoracic bio-impedance, and middle cerebral artery transcranial Doppler (TCD) blood flow velocity. *Model performance was tested by quantifying the predictions with three methods*: sensitivity and specificity, absolute error, and quantification of the log odds ratio of class 2 vs. class 0 probability estimates.

**Results:** The combination with maximal sensitivity and specificity for classes 1 and 2 was found for the model comprising volumetric features (class 1: 0.73–0.98 and class 2: 0.56–0.96). Overall lowest model error was found for the models comprising TCD curve hemodynamics. Using probability estimates the best combination of sensitivity for class 1 (0.67) and specificity (0.87) was found for the model that contained the TCD cerebral blood flow velocity derived pulse height. The highest combination for class 2 was found for the model with the volumetric features (0.72 and 0.91).

**Conclusion:** The most sensitive models for the detection of advanced CBV reduction comprised data that describe features from volumetric parameters and from cerebral blood flow velocity hemodynamics. In a validated model of hemorrhage in humans these parameters provide the best indication of the progression of central hypovolemia.

## Introduction

Hypovolemic shock is the hemodynamic response to a critically reduced central blood volume (CBV) and its diagnosis has challenged clinicians since the Second World War (Grant and Reeve, 1941; Secher and Van Lieshout, 2016). The main treatment consists of intravenous volume administration (Secher and Van Lieshout, 2005) to raise cardiac output (CO) and improve microvascular blood flow (Vincent and De Backer, 2013; Perner and De Backer, 2014; Secher and Van Lieshout, 2016) and tissue oxygen delivery (Zollei et al., 2013; Simon et al., 2015). However, detection of a clinically relevant blood volume deficit remains difficult (Marik et al., 2011; Vincent and De Backer, 2013; Bronzwaer et al., 2015; Secher and Van Lieshout, 2016) because the blood volume is not only characterized by its magnitude but also by its function as preload to the heart (Marik et al., 2011; Bronzwaer et al., 2015; Secher and Van Lieshout, 2016). From that perspective, a functional definition of “normovolemia” is by its ability to provide the heart with an adequate CBV i.e., cardiac preload that maintains stroke volume, cardiac output, and oxygen delivery (Harms et al., 2007; Truijen et al., 2010). Direct measures of CBV are not routinely available in the clinical environments of intensive care and operating room. As a result, volume treatment during anesthesia is generally planned according to a somewhat arbitrary fixed volume regime (Bundgaard-Nielsen et al., 2009) or guided by blood pressure (BP) and heart rate (HR). However, interpretation of BP and HR changes in response to a reduction in CBV is not straightforward since loss of 1 l of blood or fluid is not reflected in changes in BP (Harms et al., 2003). Therefore, optimization of tissue oxygen delivery cannot be conducted by monitoring arterial pressure alone (Michard and Teboul, 2002; Convertino, 2012; Secher, 2015; Cannesson, 2016). It is problematic that present hemodynamic monitoring techniques do not allow detection and therefore early treatment of a volume deficit before worsening of the cardio-cerebrovascular condition compromising oxygenation of the brain (Secher and Van Lieshout, 2005).

We hypothesized that the arterial pressure and transcranial cerebral blood flow velocity waveforms contain subtle information on the actual cardio-cerebrovascular condition that is hard to interpret by human visual inspection. We set out to investigate whether a machine learning model (Deo, 2015) could be trained to detect hypovolemia using hemodynamic signals during progressive reduction of CBV. This would allow determination to what extent the cardiovascular system can compensate hypovolemia, i.e., its compensatory reserve prior to (impending) circulatory collapse (Convertino et al., 2016), by classifying patients according to their actual need of fluid therapy (Convertino and Sawka, 2017) and allow timely clinical intervention. Given that the brain is highly susceptible to hypoperfusion and hypoxia we hypothesized that the cerebral flow velocity wave shape may disclose early alterations that can be alleged to the hypovolemia induced onset of cerebral hypoperfusion resulting in pre-syncope. Earlier machine learning approaches based on BP waveforms (Moulton et al., 2013) and beat-to-beat parameters (Bennis et al., 2017) showed that it can detect a reduction in CBV. To that purpose, we parametrized both the BP and TCD waveforms to make information about curve dynamics available for statistical modeling during progressive hemorrhagic shock and compared the BP features to features from other non-invasive hemodynamic technologies. We trained a model to recognize progressive hypovolemia by means of supervised machine learning and tested it on a human model of progressive hemorrhagic shock (lower body negative pressure, LBNP). The goal was to create a model that picks up on changing physiology during the transitional phase from compensated to uncompensated circulatory shock by classifying each heartbeat based on its accompanying feature information and to check which non-invasive hemodynamic monitor contributes the most sensitive information to solve this problem.

## Methods

### Subjects

Forty-two young, healthy volunteers [27 female; age: mean (SD): 24 (4) years] with no history of fainting and/or cardiac arrhythmia nor taking cardiovascular medication participated in the study. They abstained from heavy exercise and caffeinated beverages at least 12 h prior to the experiment. Before inclusion subjects underwent a medical screening prior to the experiment consisting of a medical interview, a physical examination and a 12-lead ECG. The experiments were conducted in a quiet, temperature-controlled laboratory (20–22°C). This study was carried out in accordance with the recommendations of Academic Medical Centre Amsterdam medical ethical committee (#2014_310) with written informed consent from all subjects. All subjects gave written informed consent in accordance with the Declaration of Helsinki. The protocol was approved by the medical ethical committee of the Academic Medical Centre, Amsterdam.

### Experimental protocol

Measurements were performed with subjects in the supine position. Following instrumentation, the lower body was positioned inside a lower body negative pressure (LBNP) box (Dr. Kaiser Medizintechnik, Bad Hersfeld, Germany) and sealed at the level of the iliac crest (Goswami et al., 2009). To prevent a downward shift of the body into the LBNP box disrupting the airtight sealing with loss of sub-atmospheric pressure, the LBNP box was equipped with a saddle (Bronzwaer et al., 2017a). Subjects rested for 30 min of which the final 10 were designated as baseline segment, followed by application of a single step continuous negative pressure (50 mmHg below atmospheric pressure) to the lower part of the body. The pressure inside the box was manually controlled and established within less than 20 s.

During the experiment, subjects were instructed to breathe normally and to avoid movement and muscle flexing. In compliance with our laboratory safety guidelines LBNP was terminated in case of (pre-)syncopal symptoms including sweating, light-headedness, nausea, blurred vision, and/or signs meeting one or more of the following criteria: systolic arterial pressure (SAP) below 80 mmHg, or rapid drop in BP [SAP by ≥25 mmHg·min^−1^, diastolic arterial pressure (DAP) by ≥15 mmHg·min^−1^], and drop in HR by ≥15 bpm·min^−1^. If none of these criteria occurred within 30 min, the protocol was ended. The subjects were continuously monitored by an investigator experienced in human studies and unoccupied by experimental obligations.

### Measurements

Continuous arterial BP was measured non-invasively by volume-clamp finger plethysmography with the cuff placed around the middle phalanx of the left hand placed at heart level (Nexfin, Edwards Lifesciences BMEYE, the Netherlands) and sampled at 200 Hz. Left ventricular stroke volume (SV) was determined by a pulse contour method (Nexfin CO-trek, Edwards Lifesciences BMEYE, Amsterdam, the Netherlands). Cardiac output (CO) was calculated as the SV times HR and total peripheral resistance (TPR) was the ratio of mean arterial pressure (MAP) to CO. Changes in CBV were monitored using thoracic impedance (TI) (Nihon Kohden, AI-601G, Japan) (Krantz et al., 2000; van Lieshout et al., 2005). Cerebral blood flow velocity was measured in the proximal segment of the middle cerebral artery (MCA) by means of TCD (DWL Multidop X4, Sipplingen, Germany). The left MCA was insonated through the temporal window just above the zygomatic arch at a depth of 40–60 mm with a pulsed 2 MHz probe. After signal optimization, the probe was fixed on a specially designed head-band (Marc 600, Spencer Technologies, Redmond, Washington, USA). Changes in oxygenated and deoxygenated hemoglobin (Hb) as well as their summation were measured using continuous wave near-infrared spectroscopy (NIRS) (Oxymon, Artinis, Zetten, The Netherlands). NIRS tracks cortical cerebral oxygenation during manipulation of CBF in parallel with the brain capillary oxygen saturation (Rasmussen et al., 2007). A differential path length factor was computed according to Gersten et al. (Gersten, 2015) to account for the scattering of light in the brain tissue. NIRS signals were recorded at 10 Hz. Optodes were fixed just above the supraorbital ridge and below the hairline. Changes in cutaneous perfusion may interfere with the accuracy of cerebral oximetry, therefore the distance between the transmitter and the receivers was 5 cm to assure deep enough penetration of the near-infrared light into the brain to exclude substantial contamination from the extra-cerebral circulation (Claassen et al., 2006).

End-tidal CO_2_ partial pressure (ETCO_2_) was measured through a nasal cannula connected to a capnograph (Hewlett Packard 7834A, Wokingham, UK). All recorded signals were analyzed offline (Matlab R2007b, Mathworks Inc. MA, USA) and visually inspected for artifacts and noise. Invalid beats were manually removed and interpolated.

## Modeling approach

Models were created by means of a support vector machine algorithm [libsvm software package for Matlab (Chang and Lin, 2011)]. We used a supervised learning approach to detect worsening of the cardio-cerebrovascular condition from cardiovascular stability at rest toward instability when approaching pre-syncope. To this extent, we defined three distinct classes of the hemodynamic condition (see “class definition”). The algorithm then used one of 9 designated feature sets (listed next) to detect patterns in an attempt to classify each heartbeat in one of the three classes. For each feature set a model was computed using a non-linear radial basis function (Gaussian) kernel (Bishop, 2006). To find the optimal model configuration for each respective feature set we used 64 combinations of values for both kernel width (gamma) and C-value (8 values for each). Using a randomly selected 30 subjects train vs. 1 test subject approach, this analysis was deemed optimal once the sum of sensitivity and specificity was maximal on average for all tested subjects.

### Class definition

Baseline rest as well as onset of LBNP and pre-syncope were marked. Time points originating from data during baseline were designated as class 0, samples from data during the first 75% of LBNP as class 1 and samples belonging to the last 25% of LBNP before onset of pre-syncope (i.e., end-stage LBNP) were defined as class 2 (Figure 2). Multiclass in libsvm is handled by a one-vs.-one approach (Hsu, 2002). The corresponding feature values at these time points were labeled with one of these three classes. Static features were extracted on a beat-to-beat basis whereas dynamic features (variation and trends over time) were extracted by a moving windowing function of fixed size (see model specifications) where each moved window was classified as one of three classes. Due to how the class definitions were created, class distribution was not homogenous. Around 33% of the dataset was baseline data (class 0); 50% was class 1 and 18% class 2.

### Feature extraction

**Figure 1:**
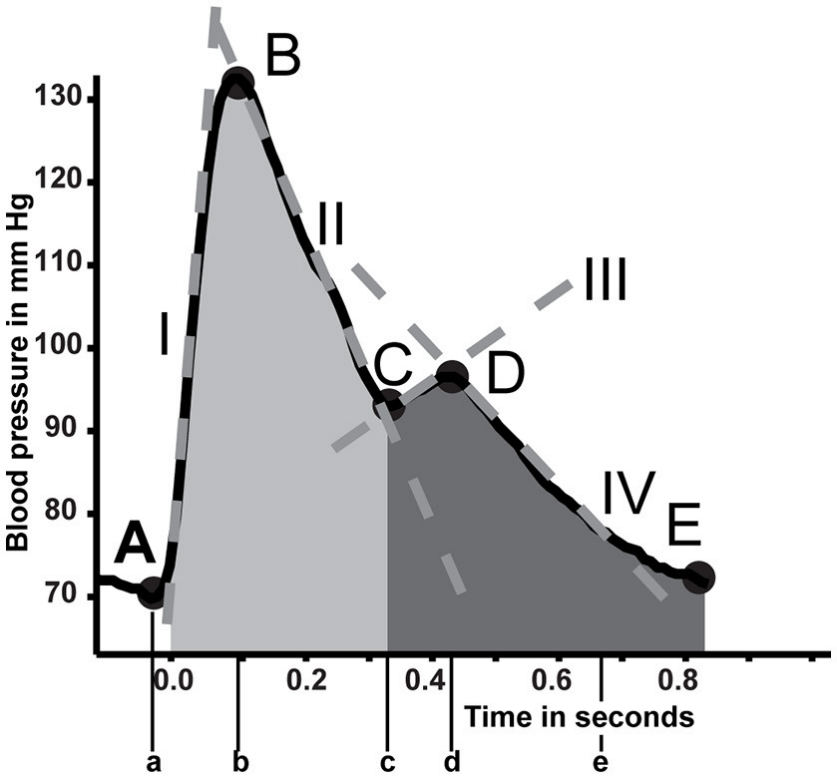
In depth analysis of the blood pressure curve. Five primary points are detected (A to E). From these points several parameters are estimated (Table A1 in Supplementary material). Positions on the curve are indicated with capital letters A through E. Their accompanying time points are described with lower case letters. Tangent lines are described with roman numerals. Areas of interest are shaded.

To test the viability of different measured parameters from non-invasive measurement modalities we designed 7 models (named model #1 through #7). All shared the BP curve dynamics features (model #1, Figure 1, Table A1 in Supplementary material). Features were then appended for models #2 through #7 for each investigated measurement modality to evaluate predictive capability when adding features from ETCO_2_, TI, NIRS, or TCD in modeling impending pre-syncope. All extracted features were down sampled by a factor 10 to abridge calculation time. Two models (named models #8 and #9) stand on their own and do not include the BP curve dynamics feature set.

**Figure 2:**
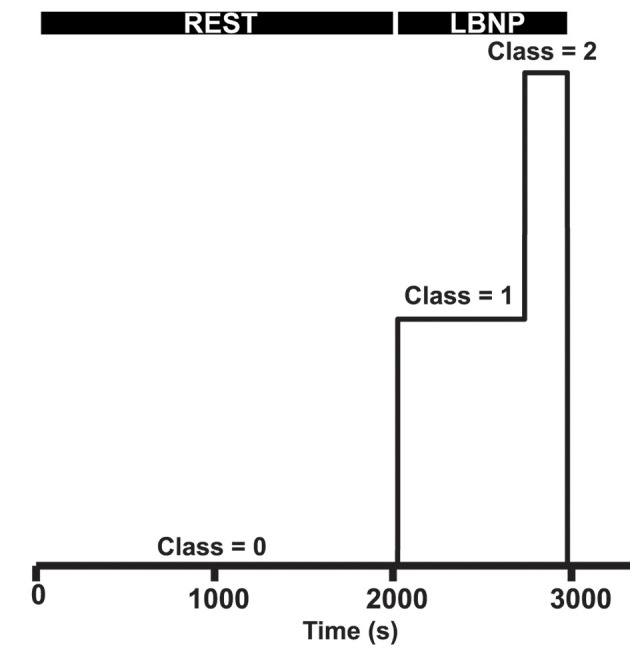
Class definitions. The first part of the measurement is defined as baseline rest (class 0), LBNP is defined as class 1, of which the last 25% is defined as end-stage LBNP before pre-syncope (class 2).

## Default model: BP curve dynamics (model #1)

From the arterial BP wave, beat-to-beat systolic, diastolic, mean, pulse pressure (SAP, DAP, MAP, and PP), interbeat interval (IBI), HR, stroke volume (SV), cardiac output (CO), left ventricular ejection time (LVET), and TPR were extracted (10 features). Four incrementally sized intervals during LBNP (30, 60, 90, and 120 s) were used for calculating trends and variances of SAP, DAP, HR, PP, and SV [4 intervals times 5 parameters for 2 techniques (trend and variation) delivers 40 features]. Additional information from the BP wave shape was extracted by wave segmentation and parametrization (Figure 1 and Table A1, Appendix Supplementary material, 15 features) making a total of 65 parameters available for the BP curve dynamics model.

## Incremental models

Either beat-to-beat interpolated ETCO_2_ partial pressure or TI were appended in models #2 and #3 respectively (each has 1 additional feature). Features extracted from the NIRS consisted of the three concentrations of Hb: oxygenated, deoxygenated, and their summation (total Hb). Ratios of oxygenated and deoxygenated to total Hb were added as well to this model (model #4, 5 additional features).

Similar to the BP wave parametrization, the same points, durations, tangents, and surface areas were derived from the cerebral blood flow velocity wave. Further features comprised systolic, diastolic, and mean flow velocity as well as the difference between systolic and diastolic flow velocity (flow velocity pulse height) and their variation and trends over the same intervals as described for model #1. Also included were the cerebral autoregulatory computed gain and phase expressed as the transfer function between MAP and MFV over a 3-min moving window between BP and MFV (Zhang et al., 1998). The low frequency band (0.06–0.15 Hz) where covariation in both signals was significant (coherence of at least 0.5) was averaged to get respective gain and phase. Model #5 will further be referred to as flow velocity curve dynamics model (FV curve dynamics). Model 6 and 7 had a single FV derived feature addition. Either the MFV or flow velocity pulse height were appended to models #6 and #7, respectively.

## Further models (models #8 and #9)

Two separate models were created to check model performance without newly introduced features. A model with the basic hemodynamic output from the Nexfin device (SAP, DAP, MAP, PP, IBI, HR, SV, CO, TPR, and LVET, model #8) was created to evaluate their additional value compared to BP and HR. A model comprising of mere BP (SAP, DAP, and MAP) and HR was introduced as a basic model (#9).

The number of features in each model is summarized in Table 1.

**Table 1:** Model description, numbering, and feature count.

**Model names and numbers**	**Amount of features (on top of base)**
#1 BP curve dynamics	65: Basic hemodynamics (10 features), curve dynamics (15 features) and trends and varations (40 features)
#2 ETCO_2_	66 (model#1 + ETCO_2_)
#3 TI	66 (model#1 + TI)
#4 NIRS	70 (model#1 +oxygenation parameters (5 features))
#5 TCD curve dynamics	125 (model#1 +60: TCD trends and variation, cerebral autoregulation)
#6 Mean MCAv	66 (model#1 +mean TCD MCAv)
#7 MCAv Pulse height	66 (model#1 +TCD pulse height)
#8 Volumetric	10: Basic hemodynamics (10 features)
#9 HR and BP	4: Systolic, diastolic, mean pressures and heart rate.

*Models 2 through 7 contain the features from model #1 with device specific features. Models 8 and 9 are smaller models, that contain features that are currently clinically used and/or available*.

Parameters were transposed into a feature matrix, normalized with respect to values during baseline and scaled so that all features ranged between 0 and 1. Alongside, a corresponding label vector that contained the appointed class per subject of each feature row was appended.

### Training and testing process

Integral data sets of subjects were included in the modeling algorithm in order to prevent contaminating data from subjects in both training and testing set. Training data consisted of data from a subselection of 30 randomly chosen subjects which changed each iteration. The resulting model was then tested on a single subject who was not part of the training set. This process was repeated for all 42 subjects. The subset of 30 subjects was chosen to reduce total training time.

### Model selection

Classification success was defined as to what extent a model correctly classifies individual samples. Each successive feature addition returned a unique classification outcome that in- or decreased model performance. Each model estimated the probability of a new sample belonging to each of the three classes. Since the classes were defined arbitrarily it is unlikely that the trained models describe a relevant physiological paradigm. To select the best model (and thus its corresponding feature set) three methods were used to quantify model performance.

1. Actual model sensitivity and specificity
  Sensitivity and specificity per class were the numbers as classified by the trained models without taking into account additional detail of probability estimates of each class. Sensitivity and specificity were calculated on a 1-vs.-all manner.
2. Individual model error
  Model error was expressed as the difference between the predefined classes and the moving average of the prediction of each model.
3. Specificity and sensitivity by accounting for probability estimates
  Next to each model classifying every individual sample, all models return a probability for the sample belonging to each respective class. In method 1 the class with the highest probability is selected as the prediction of the model for that sample. To account for probability estimates we took the ratio of the probability of a sample belonging to class 2 over its probability belonging to class 0. The logarithm of this (odds) ratio was taken and lower and upper cutoff values for this ratio were determined by using stepwise incremental thresholds to distinguish between classes 0, 1, and 2. The cutoffs were defined as optimal when the sum of both sensitivity and specificity was maximal.

## Results

The results of the search for the optimal C and gamma values per model are given in Table 2. These optimal models were chosen to compute both sensitivity and specificity (Table 3), the model errors (Table 4) and to detect optimal cutoffs for the probability estimate analysis (Table 5).

**Table 2:** Optimal model configuration.

**Feature Set**	**C**	**Gamma**
#1 BP curve dynamics	0.13895	0.002683
#2 BP curve dynamics & ETCO_2_	0.13895	0.051795
#3 BP curve dynamics & TI	0.13895	0.051795
#4 BP curve dynamics & NIRS	0.517947	0.007197
#5 BP curve dynamics & TCD	100	0.019307
#6 BP curve dynamics & MCAv mean	0.037276	0.13895
#7 BP curve dynamics & MCAv pulse height	7.196857	0.001
#8 Volumetric	0.0100	0.001
#9 HR and BP	0.5179	0.0027

*Optimal results following the 64-fold optimization steps for different incremental values for regularization parameter C (misclassification penalty) and gamma (deviation of the radial basis Kernel) for each feature set*.

**Table 3:** Median [25% 75%] sensitivity and specificity for different features sets for the three designated classes.

**Feature set**	**Sensitivity**			**Specificity**		
	**Class 0**	**Class 1**	**Class 2**	**Class 0**	**Class 1**	**Class 2**
#1 BP curve dynamics	0.99 [0.98; 0.99]	0.63 [0.54; 0.72]	0.56 [0.37; 0.76]	0.81 [0.75; 0.87]	0.98 [0.93; 0.99]	0.95 [0.92; 0.97]
#2 BP curve dynamics & ETCO_2_	0.99 [0.98; 0.99]	0.62 [0.50; 0.72]	0.53 [0.31; 0.69]	0.81 [0.71; 0.85]	0.96 [0.93; 0.98]	0.96 [0.93; 0.98]
#3 BP curve dynamics & TI	0.99 [0.98; 0.99]	0.63 [0.54; 0.73]	0.51 [0.27; 0.69]	0.81 [0.74; 0.88]	0.96 [0.93; 0.98]	0.96 [0.93; 0.98]
#4 BP curve dynamics & NIRS	0.99 [0.98; 0.99]	0.64 [0.55; 0.70]	0.53 [0.35; 0.64]	0.81 [0.74; 0.90]	0.97 [0.93; 0.98]	0.96 [0.93; 0.97]
#5 BP curve dynamics & TCD	0.99 [0.99; 1.00]	0.58 [0.48; 0.66]	0.47 [0.26; 0.61]	0.72 [0.62; 0.83]	0.98 [0.90; 0.99]	0.96 [0.93; 0.98]
#6 BP curve dynamics & MCAv mean	0.99 [0.98; 0.99]	0.63 [0.54; 0.71]	0.50 [0.29; 0.69]	0.80 [0.73; 0.88]	0.96 [0.92; 0.98]	0.96 [0.93; 0.97]
#7 BP curve dynamics & MCAv Pulse height	0.99 [0.98; 0.99]	0.62 [0.57; 0.69]	0.52 [0.28; 0.71]	0.81 [0.72; 0.88]	0.97 [0.91; 0.98]	0.96 [0.91; 0.98]
#8 Volumetric	0.99 [0.98; 0.99]	**0.73 [0.68; 0.81]**	**0.56 [0.40; 0.77]**	0.93 [0.88; 0.97]	**0.98 [0.91; 0.99]**	**0.96 [0.93; 0.97]**
#9 HR and BP	0.97 [0.94; 0.98]	0.62 [0.43; 0.67]	0.49 [0.20; 0.73]	0.79 [0.60; 0.89]	0.94 [0.90; 0.97]	0.95 [0.92; 0.96]

*Class 0: rest; class 1: during LBNP; class 2: final stage LBNP before pre-syncope per model structure. Highest cumulative sensitivity, specificity in that class is indicated in bold*.

**Table 4:** Median mean squared errors per model.

**Class/Model**	**Class 0**	**Class 1**	**Class 2**	**Total error**
#1 BP curve dynamics	0.11	**0.06**	0.82	1
#2 ETCO^2^	0.13	0.12	0.65	0.89
#3 TI	0.11	0.11	0.67	0.89
#4 NIRS	0.1	0.11	0.74	0.95
#5 FV curve dynamics	0.19	**0.06**	**0.53**	**0.78**
#6 MCAv mean	0.11	0.1	0.7	0.91
#7 MCAv PP	0.11	0.11	0.81	1.03
#8 Volumetric	**0.03**	0.07	0.71	0.82
#9 HR and BP	0.12	0.16	0.81	1.09

**Figure 3:**
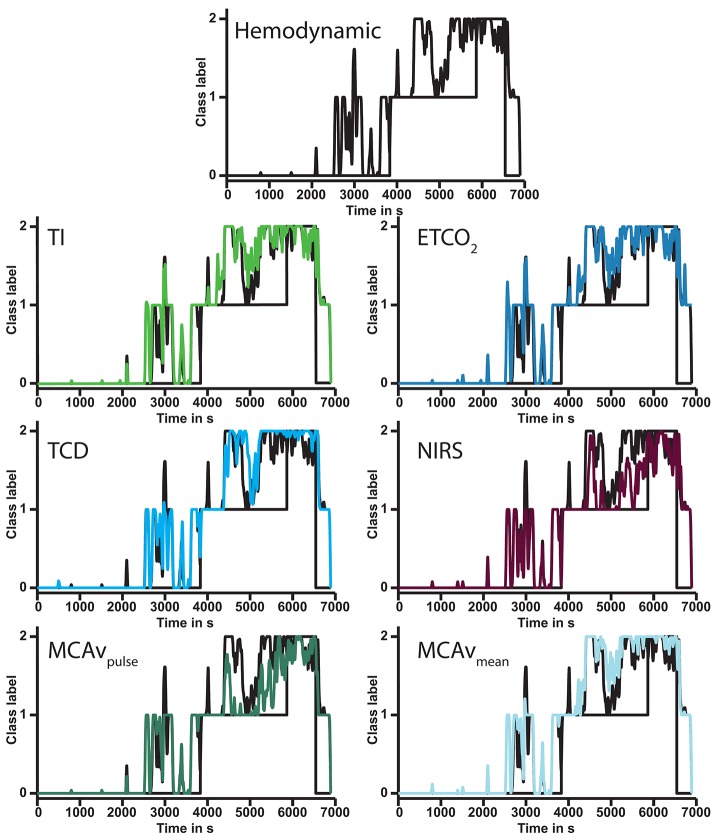
Output of six models compared to BP curve dynamics model (#1, **top**) in a single subject. Each subsequent graph shows the modulation of the addition of the annotated feature(s). In this subject the model for MCAv pulse height (**bottom left**) had the lowest error. Note that all model outputs increase with increasing duration of lower body negative pressure. ETCO_2_, end-tidal carbon dioxide pressure; TI, thoracic impedance; NIRS, near infrared spectroscopy; TCD, transcranial Doppler; MCAv, middle cerebral artery velocity; MCAv_pulse_, middle cerebral artery velocity pulse height.

*Expressed as difference between moving averaged prediction and the predefined class line (Figure 3). Lowest error per class indicated in bold*.

**Table 5:** Sensitivities and specificities of all models using two cutoffs on probability estimates.

**Model NR**	**Class 0**		**Class 1**		**Class 2**		**Cutoffs**	
	**Sensitivity**	**Specificity**	**Sensitivity**	**Specificity**	**Sensitivity**	**Specificity**	**low**	**high**
1	0.9047	0.9310	0.5453	0.8942	0.7301	0.9012	−1.01	7.19
2	0.8984	0.9310	0.5985	0.8803	0.6835	0.9181	−1.45	7.94
3	0.8980	0.9334	0.5886	0.8815	0.6952	0.9144	−1.51	7.79
4	0.8872	0.9421	0.6208	0.8687	0.6666	0.9199	−1.51	7.79
5	0.9457	0.9252	0.6272	0.9130	0.6066	0.9289	1.09	7.58
6	0.8898	0.9326	0.6351	0.8701	0.6469	0.9258	−1.28	8.01
7	0.9082	0.9341	**0.6688**	**0.8779**	0.5981	0.9370	−1.31	8.19
8	**0.9536**	**0.9562**	0.6007	0.9325	**0.7239**	**0.9114**	−1.52	8.17
9	0.8934	0.9000	0.6092	0.8697	0.6064	0.9298	−1.25	5.34

*Model numbers indicate: 1, BP curve dynamics; 2, ETCO_2_; 3, TI; 4, NIRS; 5, TCD dynamics; 6, MCAv mean; 7, MCAv pulse height; 8, Volumetric; 9, HR and BP. Bold: highest cumulative sensitivity, specificity in that class*.

### Actual model sensitivity and specificity

Regarding classes 1 and 2, the combination with highest sensitivity and specificity was found for the model comprising volumetric features (#8) (class 1: sensitivity: 0.73; specificity: 0.98; class 2: sensitivity: 0.56; specificity: 0.96) (Table 3). Adding variation, trends and BP curve dynamics (model #1, Figure 1) did not improve the performance of the model for classes 1 (sensitivity 0.63; specificity 0.98) and 2 (sensitivity 0.56; specificity 0.95). Sequentially adding features of ETCO_2_, TI, or from NIRS or TCD devices also did not improve classifying actual model sensitivity. Specificity was maintained.

### Individual model error

The FV curve dynamics model (#5) had the lowest error for all three classes combined (Table 4). The median error of the BP curve dynamics (#1) vs. FV curve dynamics model (#5) was greater for class 2. The largest fraction of subjects (12/42) benefited from the FV curve dynamics model (#5) since it had the lowest overall error. Models with either mean MCAv (model # 6) or pulse height of MCAv (model #7) accounted for another 8/42 subjects. The BP curve dynamics model (#1) had the lowest error for 10/42 subjects. Models including ETCO_2_ (#2) or NIRS (#4) both performed best 5/42 times. The TI model (#3) came in last as the best model for 2/42 subjects.

### Specificity and sensitivity by accounting for probability estimates

In general, all models had similar sensitivity for baseline (class 0) (range: [0.89; 0.95]) and specificity ([0.90; 0.96]) (Table 5). Regarding class 1, the best combination of sensitivity and specificity was found for the model that contained the FV derived pulse height (model #7). The highest combination for class 2 was found for the model with the volumetric features (model #8). This model also had the highest combination for both class 1 and class 2 together. An overview of all classification samples can be found in the confusion matrices (Stehman, 1997) in Appendix 2 Supplementary material. For both the actual models and after accounting for probability estimates. In general it can be seen that the models encounter most difficulty in the distinction between class 1 and class 2 while the distinction between class 0 and either class 1 or 2 is clearer.

## Discussion

The new findings of the present study are, first, that distinguishing between normovolemia and considerable central hypovolemia in healthy young adults requires information from volumetric hemodynamic features beyond BP and HR, such as IBI, SV, CO, LVET, and TPR. Second, the cerebral blood flow velocity parameters reduced model error, possibly due to the creation of a more easily separable solution.

Features derived from the BP curve, ETCO_2_, TI, and from cerebral blood flow velocity and brain cortical oxygenation did not improve the classification in terms of sensitivity to detect advanced class 2 hypovolemia. In contrast, cerebral blood flow velocity models (#5–7) outperformed the other models in terms of absolute error from the predefined (artificially created) classes. Models 2–4 [ETCO_2_, central blood volume (TI), and cerebral cortical oxygenation (NIRS)] contributed to such an extent that they were the best discriminative model for fewer subjects and therefore in general seem less sensitive to the detection of CBV depletion.

In machine learning or datamining approaches large datasets are investigated to determine whether these features together result in a better solution to the problem at hand. A mechanistic approach may not find such a solution in a multidimensional space. The underlying physiological mechanisms can ideally be described by such a mechanistic approach so that it can explain the wide variety of pathophysiology as is seen in different patients. Unfortunately, this is not easily achieved and assumptions would have to be made for many parameters, as they cannot be measured in real time (or at all) resulting in a model that is not very useful for individual cases. Due to the large natural variation between subjects, some individuals increase peripheral resistance to maintain adequate blood pressure, whereas others increase heart rate at onset of LBNP, yet another group responds in a mixed fashion. We do not think these subtleties can be grasped by a mechanistic approach, unless the responses of a patient would be assessed beforehand which is not feasible in clinical practice.

It is possible that a unique set of features exists from different devices that gives an even better solution. To assess this possibility would require a feature selection process which is cumbersome for this amount of models. We considered that these devices are either connected as monitors to patients or not. If so, they return a fixed array of features which was included in the models here. This study therefore aimed to describe which monitors deliver the most sensitive features and should therefore be connected as a monitor for detecting changing CBV.

### Limitations

By design the subjects were healthy individuals exposed to simulated bleeding which restrains us from extrapolating the data to elderly subjects, considering that with healthy aging brain perfusion becomes increasingly dependent on cardiac output (Bronzwaer et al., 2017b).

The current models require that its features are normalized to a reference baseline condition. This will be required as well for future use of the models. Future studies should therefore be directed at finding similar model accuracy without baseline normalization. We recognize that eliminating normalization will increase intersubject scatter, inevitably reducing classification performance.

We consider the possibility that adding a considerable number of features introduced the phenomenon known as overfitting. This would imply that the model is being too specifically trained on training data and may not function equally well on new data. Since the SVM method is a regularization model, the introduction of large amounts of features does not necessarily have to lead to worse performance due to overfitting. However, we selected optimal gamma and C on the held-out data, which could have led to a form of overfitting, but due to the newly random selection of 30 subjects in the testing step as well, this is expected to be marginal.

Classes were not distributed homogenously. Especially during training this could have had a significant effect on the outcome as the algorithm could have had relatively more examples of what is considered class 2 with respect to the other classes.

Since the training was performed on a subset of subject data, the reported numbers for sensitivity and specificity are not absolute and will be different if the analysis is repeated. In healthy subjects, variation in cardiovascular responses to sympathetic stimulation evoked by submaximal lower body negative pressure (LBNP) is considerable (Bronzwaer et al., 2016). Differences in resting HR between subjects suggest individually programmed reflex strategies of autonomic blood pressure control which may contribute to the hitherto unpredictable variance observed in cardiovascular reflex responses to central hypovolemia (Bronzwaer et al., 2016). Due to this large natural variation in subject responses we considered that by using a random subset the models are not focused on a fixed set but will vary with each iteration. Also since not everyone experiences symptoms of pre-syncope in the exact same way there may be a bias toward the point that was defined as pre-syncope here. By using a random subset of individuals the models were never trained on the full set of this bias but included different subjects each training iteration.

### Classification and tracking

The fact that feature sets from cerebral oxygenation, central blood volume, or cerebral blood flow velocity data do not qualify beats better than the volumetric features seems to suggest that their capability to predict pre-syncope may be low or at least not better than HR and BP combined with LVET, CO, TPR, and SV. However, the probability estimation of class 2 shows a notable increase indicating that in the large majority of subjects the developed models all recognized the process of moving from baseline, to CBV depleted, to pre-syncope.

One explanation for the limited difference in performance between models #1 and #8 may be that the Nexfin built-in algorithms in itself include a BP wave shape analysis (pulse contour).

Any attempt to produce a complete clinical classification of hemorrhagic shock for the individual patient can be only provisional due to the complex interrelations in physiological adaptive responses (McMichael, 1944; Michard and Teboul, 2002; Perner and De Backer, 2014). Similarly, between healthy subjects the variation in cardiovascular responses to sympathetic stimulation evoked by bleeding is considerable. Distinct cardiovascular response patterns of preferential autonomic blood pressure control appear consistent over time within one subject but with considerable inter-individual variance in tolerance to hypovolemia (Convertino et al., 2012; Ryan et al., 2012; Bronzwaer et al., 2016). This explains the difference in time until pre-syncope and thus differences in the number of samples between subjects available to the models (Jellema et al., 1996). The models are nevertheless requested to assign one of the three classes to each individual subject through the whole trajectory from normo- to hypovolemia. Also, the large number of samples available for class 0 compared to class 1 and 2 creates an unequal distribution of samples between the three classes. This also explains the overall high specificity, since classification of a sample *not* belonging to the investigated class could mean either of two remaining classes.

The translation from model output to underlying physiological events is by no means straightforward. Defining the classes from normo- to hypovolemia served merely to create an artificial distinction between the ongoing circulatory adaptive responses to progressive central hypovolemia. As a consequence, the underlying physiological adaptive responses may not fit into the predefined classes and reported sensitivity does neither reflect direct classification of physiology. However, the actual sensitivity/specificity is amenable for improvement by using the certitude of the model by introducing a cut-off analysis on the probability estimates as proposed in order to quantify model performance. This better approaches a classification on a physiological response as changing probabilities of the classes could hint at progression toward cardiovascular instability respectively a return to normovolemia that can be tracked over time.

Ideally, model performance is described by the individual (moving averaged) prediction line as they tend to increase during progressive hypovolemia (Figure 3), as a visual manifestation of the increasing probability of impending circulatory collapse since it immediately visualizes into what direction the patient's hemodynamic condition is headed. We attempted to overcome the fact that this measure is difficult to express as a numeric error by implementing three different ways of model performance quantification. This probability estimate analysis increased model sensitivity and specificity by taking into account the complexity of the output of the model in the relative large variation of subject responses to hypovolemia.

Classification of heart beats belonging to either class 0 or class 1 and 2 is straightforward, and appeared linearly separable using only a few features. This may be due to the fact that this protocol was executed in a controlled setting and due to the fact that the data was normalized to a baseline value. Detecting whether a particular beat should be classified to either the class 1 or class 2 state of being hypovolemic is more challenging, hence the use of a non-linear Gaussian kernel. Due to the large inter-individual variance and artificial nature of class creation, the data show a considerable overlap for the currently presented features, which hindered us into constructing models with a higher sensitivity. Rather, the moving average during the classification process in itself has the potential to function as a real-time visualization of progress toward hypovolemia induced cardiovascular instability.

## Author contributions

Data acquisition: BvdS, FB, WS. Analysis: BvdS. Figure preparation: BvdS, JvL. Manuscript drafting: BvdS, JvL. Data interpretation: BvdS, FB, TD, BW, WS, JvL. Manuscript editing: BvdS, BW, FB, TD, WS, JvL.

### Conflict of interest statement

This work was supported by an educational grant from Edwards Lifesciences. They, however had no say in any of the content provided or the direction of submitted research.

## References

[B1] Bennis F. C. Van der Ster B. Van Lieshout J. Andriessen P. Delhaas T. (2017). A machine-learning based analysis for the recognition of progressive central hypovolemia. Physiol. Meas. 38, 1791–1801. 10.1088/1361-6579/aa7d3d 28671554

[B2] Bishop M. C. (2006). Pattern Recognition and Machine Learning. Cambridge: Springer Science+Business Medica, LLC.

[B3] Bronzwaer A. G. Verbree J. Stok W. J. Daemen M. J. van Buchem M. A. van Osch M. J.. (2017a). The cerebrovascular response to lower-body negative pressure vs. head-up tilt. J. Appl. Physiol. (1985) 122, 877–883. 10.1152/japplphysiol.00797.2016 28082333

[B4] Bronzwaer A. Verbree J. Stok W. J. Daemen M. J. A. P. van Buchem M. A. van Osch M. J. P.. (2017b). Aging modifies the effect of cardiac output on middle cerebral artery blood flow velocity. Physiol. Rep. 5:e13361. 10.14814/phy2.13361 28912128 PMC5599856

[B5] Bronzwaer A. S. Ouweneel D. M. Stok W. J. Westerhof B. E. van Lieshout J. J. (2015). Arterial pressure variation as a biomarker of preload dependency in spontaneously breathing subjects - a proof of principle. PLoS ONE 10:e0137364. 10.1371/journal.pone.0137364 26335939 PMC4559442

[B6] Bronzwaer A.-S. G. T. Verbree J. Stok W. J. van Buchem M. A. Daemen M. J. A. P. van Osch M. J. P.. (2016). Cardiovascular response patterns to sympathetic stimulation by central hypovolemia. Front. Physiol. 7:235. 10.3389/fphys.2016.00235 27378944 PMC4913112

[B7] Bundgaard-Nielsen M. Secher N. H. Kehlet H. (2009). ‘Liberal’ vs. 'restrictive' perioperative fluid therapy–a critical assessment of the evidence. Acta Anaesthesiol. Scand. 53, 843–851. 10.1111/j.1399-6576.2009.02029.x 19519723

[B8] Cannesson M. (2016). Non-invasive guidance of fluid therapy, in Clinical Fluid Therapy in the Perioperative Setting, 2nd Edn, ed Hahn R. G. (Cambridge: Cambridge University Press), 120–127.

[B9] Chang C.-C. Lin C.-J. (2011). {LIBSVM}: a library for support vector machines. ACM Trans. Intell. Syst. Technol. 2, 21–27. 10.1145/1961189.1961199

[B10] Claassen J. A. Colier W. N. Jansen R. W. (2006). Reproducibility of cerebral blood volume measurements by near infrared spectroscopy in 16 healthy elderly subjects. Physiol. Meas. 27, 255–264. 10.1088/0967-3334/27/3/004 16462012

[B11] Convertino V. A. (2012). Blood pressure measurement for accurate assessment of patient status in emergency medical settings. Aviat. Space Environ. Med. 83, 614–619. 10.3357/asem.3204.2012 22764618

[B12] Convertino V. A. Sawka M. N. (2017). Wearable technology for compensatory reserve to sense hypovolemia. J. Appl. Physiol. (1985). [Epub ahead of print]. 10.1152/japplphysiol.00264.2017 28751369

[B13] Convertino V. A. Rickards C. A. Ryan K. L. (2012). Autonomic mechanisms associated with heart rate and vasoconstrictor reserves. Clin. Auton. Res. 22, 123–130. 10.1007/s10286-011-0151-5 22083580

[B14] Convertino V. A. Wirt M. D. Glenn J. F. Lein B. C. (2016). The compensatory reserve for early and accurate prediction of hemodynamic compromise: a review of the underlying physiology. Shock 45, 580–590. 10.1097/shk.0000000000000559 26950588

[B15] Deo R. C. (2015). Machine learning in medicine. Circulation 132, 1920–1930. 10.1161/CIRCULATIONAHA.115.001593 26572668 PMC5831252

[B16] Gersten A. (2015). Probing brain oxygenation with Near Infrared Spectroscopy (NIRS) — The role of carbon dioxide and blood pressure. arXiv:1510.05307v1 10.5772/59113

[B17] Goswami N. Grasser E. Roessler A. Schneditz D. Hinghofer-Szalkay H. (2009). The cardiovascular response to lower body negative pressure in humans depends on seal location. Physiol. Res. 58, 311–318. 18637716 10.33549/physiolres.931431

[B18] Grant R. T. Reeve E. B. (1941). Clinical observations on air-raid casualties. Br. Med. J. 2, 293–297. 20783834 10.1136/bmj.2.4208.293 PMC2162444

[B19] Harms M. P. M. van Lieshout J. J. Jenstrup M. Pott F. Secher N. H. (2003). Postural effects on cardiac output and mixed venous oxygen saturation in humans. Exp. Physiol. 88, 611–616. 10.1113/eph8802580 12955161

[B20] Harms M. P. Secher N. H. van Lieshout J. J. (2007). Monitoring of goal-directed fluid challenge. Crit Care Med. 35, 673. 10.1097/01.CCM.0000254332.29816.C6 17251732

[B21] Hsu C.-W. (2002). A comparison of methods for multi-class support vector machines, in IEEE Transactions on Neural Networks, ed Lin C.-J. (Berlin; Heidelberg: Springer). 10.1109/72.991427 18244442

[B22] Jellema W. T. Imholz B. P. van Goudoever J. Wesseling K. H. van Lieshout J. J. (1996). Finger arterial versus intrabrachial pressure and continuous cardiac output during head-up tilt testing in healthy subjects. Clin. Sci. 91, 193–200. 8795443 10.1042/cs0910193

[B23] Krantz T. Cai Y. Lauritsen T. Warberg J. Secher N. H. (2000). Accurate monitoring of blood loss: thoracic electrical impedance during hemorrhage in the pig. Acta Anaesthesiol. Scand. 44, 598–604. 10.1034/j.1399-6576.2000.00519.x 10786749

[B24] Marik P. Monnet X. Teboul J. L. (2011). Hemodynamic parameters to guide fluid therapy. Ann. Intensive Care 1, 1–9. 10.1186/2110-5820-1-1 21906322 PMC3159904

[B25] McMichael J. (1944). Clinical aspects of shock. J. Am. Med. Assoc. 124, 275–281. 10.1001/jama.1944.02850050007003

[B26] Michard F. Teboul J. L. (2002). Predicting fluid responsiveness in ICU patients: a critical analysis of the evidence. Chest 121, 2000–2008. 10.1378/chest.121.6.2000 12065368

[B27] Moulton S. L. Mulligan J. Grudic G. Z. Convertino V. A. (2013). Running on empty? The compensatory reserve index. J. Trauma Acute Care Surg. 75, 1053–1059. 10.1097/TA.0b013e3182aa811a 24256681

[B28] Perner A. De Backer D. (2014). Understanding hypovolaemia. Intensive Care Med. 40, 613–615. 10.1007/s00134-014-3223-x 24556910

[B29] Rasmussen P. Dawson E. A. Nybo L. van Lieshout J. J. Secher N. H. Gjedde A. (2007). Capillary-oxygenation-level-dependent near-infrared spectrometry in frontal lobe of humans. J. Cereb. Blood Flow Metab. 27, 1082–1093. 10.1038/sj.jcbfm.9600416 17077816

[B30] Ryan K. L. Rickards C. A. Hinojosa-Laborde C. Cooke W. H. Convertino V. A. (2012). Sympathetic responses to central hypovolemia: new insights from microneurographic recordings. Front. Physiol. 3:110. 10.3389/fphys.2012.00110 22557974 PMC3337468

[B31] Secher N. H. (2015). Eat, drink and be merry–and protect the brain. Exp. Physiol. 100:991. 10.1113/EP085398 26331216

[B32] Secher N. H. Van Lieshout J. J. (2005). Normovolaemia defined by central blood volume and venous oxygen saturation. Clin. Exp. Pharmacol. Physiol. 32, 901–910. 10.1111/j.1440-1681.2005.04283.x 16405445

[B33] Secher N. H. Van Lieshout J. J. (2016). Hypovolemic shock, in Clinical Fluid Therapy in the Perioperative Setting, 2nd Edn, ed Hahn R. G. (Cambridge: Cambridge University Press), 222–231.

[B34] Simon J. Farkas T. Gingl Z. Csillik A. Korsos A. Rudas L.. (2015). Noninvasive continuous arterial pressure measurements in the assessment of acute, severe central hypovolemia. Acta Physiol. Hung. 102, 43–50. 10.1556/APhysiol.102.2015.1.4 25804388

[B35] Stehman S. V. (1997). Selecting and interpreting measures of thematic classification accuracy. Remote Sens. Environ. 62, 77–89. 10.1016/S0034-4257(97)00083-7

[B36] Truijen J. Bundgaard-Nielsen M. van Lieshout J. J. (2010). A definition of normovolaemia and consequences for cardiovascular control during orthostatic and environmental stress. Eur. J. Appl. Physiol. 109, 141–157. 10.1007/s00421-009-1346-5 20052592 PMC2861179

[B37] van Lieshout J. J. Harms M. P. Pott F. Jenstrup M. Secher N. H. (2005). Stroke volume of the heart and thoracic fluid content during head-up and head-down tilt in humans. Acta Anaesthesiol. Scand. 49, 1287–1292. 10.1111/j.1399-6576.2005.00841.x 16146465

[B38] Vincent J.-L. De Backer D. (2013). Circulatory Shock. N. Engl. J. Med. 369, 1726–1734. 10.1056/NEJMra1208943 24171518

[B39] Zhang R. Zuckerman J. H. Giller C. A. Levine B. D. (1998). Transfer function analysis of dynamic cerebral autoregulation in humans. Am. J. Physiol. Heart Circ. Physiol. 274(1 Pt 2), H233–H241. 9458872 10.1152/ajpheart.1998.274.1.h233

[B40] Zollei E. Bertalan V. Nemeth A. Csabi P. Laszlo I. Kaszaki J.. (2013). Non-invasive detection of hypovolemia or fluid responsiveness in spontaneously breathing subjects. BMC Anesthesiol. 13:40. 10.1186/1471-2253-13-40 24188480 PMC3829671

