# Supplementary material for: Support Vector Machine Based Monitoring of Cardio-Cerebrovascular Reserve during Simulated Hemorrhage
Source: Front Physiol. 2018 Jan 5;8:1057. doi: 10.3389/fphys.2017.01057 (PMC5761201; doi:10.3389/fphys.2017.01057)
Supplement: Supplementary file 1 [file DataSheet1.pdf]

APPENDIX 1: BP PARAMETRIZATION

TABLE A1 CURVE SPECIFIC PARAMETERS COMPUTED FROM EACH BLOOD PRESSURE WAVE

Feature name	Feature description and explanation of Figure 1	Equation
A	Position of A relative to systole	
B	Systole	-
C	Dicrotic notch; relative to systole	-
D	Position of D relative to systole	-
E	Position of E relative to systole (red dot in Figure 1)	-
a	Time point of A relative to e	-
b	Time point of B relative to e	-
c	Time point of C relative to e	-
d	Time point of D relative to e	-
tangAB	Slope between points A and B (dashed line I in Figure 1)	$y = \frac{B - A}{b - a}x + A$
tangBC	Slope between points B and C (dashed line II in Figure 1)	$y = \frac{C - B}{c - a}x + B$
tangCD	Slope between points C and D (dashed line III in Figure 1)	$y = \frac{D - C}{d - c}x + C$
tangDE	Slope between points D and E (dashed line IV in Figure 1)	$y = \frac{E - D}{e - d}x + D$
ratBC	Ratio between points B and C	$\text{ratBC} = \frac{B}{C}$
ratCD	Ratio between points B and C	$\text{ratCD} = \frac{C}{D}$
ratBD	Ratio between points B and D	$\text{ratBD} = \frac{D}{B}$
surAB	Line integral of the BP curve for time segment a to b (light area in Figure 1)	$\int_a^b f(t)$

surAE	Line integral of the entire BP curve (dark & light area in Figure 1)	$\int_a^e f(t)$
surCE	Line integral of the BP curve for time segment c to e (dark area in Figure 1)	$\int_a^c f(t)$
ratSurAB_AE	ratio of surface A to B to A to E	$\frac{\int_a^b f(t)}{\int_a^e f(t)}$
ratSurCE_AE	ratio of surface C to E to A to E	$\frac{\int_c^e f(t)}{\int_a^e f(t)}$
ratSurCE_AB	ratio of surface C to E to A to B	$\frac{\int_c^e f(t)}{\int_a^b f(t)}$
DPDTMax	Max slope of the BP curve over segment A to B	$\left[max\left(\frac{\Delta P}{dt}\right)\right]_a^b$
Height of dicrotic notch	Local minimum surrounding LVET normalized to systolic pressure.	

Table A1. Blood pressure wave shape is indicated with F(t). Detected points A through E and their corresponding times A through E are not included as features but only serve for explanation purposes.

APPENDIX 2: CONFUSION MATRICES

In this appendix the confusion matrix of both steps of the error calculation are reported. On the left is the initial accuracy for each model and on the right each model accuracy after computing the optimal cut-off values for the probability estimates. These cut-offs were defined optimal once the distinction between class 1 and class 2 was the highest. Since this was deemed clinically the most important.

Each cell has two numbers. For the first 3 rows, the absolute numbers indicated the amount of samples classified like this. The percentage in that cell indicates the proportion of the sample of this cell compared to the complete data set size it was tested on. Column and row 4 have two percentage numbers: the top one indicating the amount of correct classifications, the bottom number indicating the amount of mistakes. Horizonally these indicate the amount of times a prediction is correct. Vertically this indicates the amount of times a target class is classified correctly.

The diagonal cells show the number and percentage of correct classifications.

Model #1. BP Curve Dynamics

Initial

Predicted Class	Target class 0	Target class 1	Target class 2	
0	90394<br>56.5%	18707<br>11.7%	867<br>0.5%	82%<br>18%
1	1919<br>1.2%	31922<br>19.9%	3620<br>2.3%	85%<br>15%
2	25<br>0.0%	7962<br>5.0%	4597<br>2.9%	37%<br>63%
	98%<br>2%	54%<br>46%	51%<br>49%	79%<br>21%

optimal cut-offs

Predicted Class	Target class 0	Target class 1	Target class 2	
0	108408<br>67.7%	1136<br>0.7%	424<br>0.3%	99%<br>1%
1	15981<br>10.0%	11601<br>7.3%	9879<br>6.2%	31%<br>69%
2	1940<br>1.2%	3265<br>2.0%	7379<br>4.6%	59%<br>41%
	86%<br>14%	72%<br>28%	42%<br>58%	80%<br>20%

Model #2. $ETCO_2$

Initial

Predicted Class	Target class 0	Target class 1	Target class 2	
0	88848<br>55.5%	20011<br>12.5%	1109<br>0.7%	81%<br>19%
1	1796<br>1.1%	29622<br>18.5%	6043<br>3.8%	79%<br>21%
2	28<br>0.0%	6737<br>4.2%	5819<br>3.6%	46%<br>54%
	98%<br>2%	53%<br>47%	45%<br>55%	78%<br>22%

optimal cut-offs

Predicted Class	Target class 0	Target class 1	Target class 2	
0	107537<br>67.2%	2104<br>1.3%	327<br>0.2%	98%<br>2%
1	12443<br>7.8%	15690<br>9.8%	9328<br>5.8%	42%<br>58%
2	1199<br>0.7%	3871<br>2.4%	7514<br>4.7%	60%<br>40%
	89%<br>11%	72%<br>28%	44%<br>56%	82%<br>18%

Model #3. TI

Initial

Predicted Class				
0	90137<br>56.3%	18634<br>11.6%	1197<br>0.7%	82%<br>18%
1	1868<br>1.2%	29777<br>18.6%	5816<br>3.6%	79%<br>21%
2	27<br>0.0%	7107<br>4.4%	5450<br>3.4%	43%<br>57%
	98%<br>2%	54%<br>46%	44%<br>56%	78%<br>22%
Target class	0	1	2	

optimal cut-offs

Predicted Class				
0	107690<br>67.3%	1967<br>1.2 %	311<br>0.2%	98%<br>2%
1	13246<br>8.3%	15329<br>9.6%	8886<br>5.6%	41%<br>59%
2	1253<br>0.8%	4089<br>2.6%	7242<br>4.5%	58%<br>42%
	88%<br>12%	72%<br>28%	44%<br>56%	81%<br>19%
Target class	0	1	2	

Model #4. NIRS

Initial

Predicted Class				
0	90262<br>56.4%	18688<br>11.7%	1018<br>0.6%	82%<br>18%
1	1889<br>1.2%	31544<br>19.7%	4028<br>2.5%	84%<br>16%
2	26<br>0.0%	7236<br>4.5%	5322<br>3.3%	42%<br>58%
	98%<br>2%	55%<br>45%	51%<br>49%	79%<br>21%
Target class	0	1	2	

optimal cut-offs

Predicted Class				
0	108645<br>67.9%	1002<br>0.6%	321<br>0.2%	99%<br>1%
1	16705<br>10.4%	11812<br>7.4%	8944<br>5.6%	32%<br>68%
2	1844<br>1.2%	3416<br>2.1%	7324<br>4.6%	58%<br>42%
	85%<br>15%	73%<br>27%	44%<br>56%	80%<br>20%
Target class	0	1	2	

Model #5. FV curve dynamics

Initial

Predicted Class				
0	76658<br>47.9%	31988<br>20.0%	1322<br>0.8%	70%<br>30%
1	554<br>0.3%	30549<br>19.1%	6358<br>4.0%	82%<br>18%
2	20<br>0.0%	6245<br>3.9%	6319<br>3.9%	50%<br>50%
	99%<br>1%	44%<br>56%	45%<br>55%	71%<br>29%
Target class	0	1	2	

optimal cut-offs

Predicted Class				
0	109143<br>68.2%	695<br>0.4%	130<br>0.1%	99%<br>1%
1	15157<br>9.5%	13018<br>8.1%	9286<br>5.8%	35%<br>65%
2	2044<br>1.3%	3373<br>2.1%	7167<br>4.5%	57%<br>43%
	86%<br>14%	76%<br>24%	43%<br>57%	81%<br>19%
Target class	0	1	2	

Model #6. MCAv mean

Initial

Predicted Class	Target class 0	Target class 1	Target class 2	
0	89663<br>56.0%	18869<br>11.8%	1436<br>0.9%	82%<br>18%
1	1886<br>0.3%	29820<br>18.6%	5755<br>3.6%	80%<br>20%
2	28<br>0.0%	6646<br>4.2%	5910<br>3.7%	47%<br>53%
	98%<br>2%	54%<br>46%	45%<br>55%	78%<br>22%

optimal cut-offs

Predicted Class	Target class 0	Target class 1	Target class 2	
0	108050<br>67.5%	1410<br>0.9%	508<br>0.3%	98%<br>2%
1	17274<br>10.8%	11622<br>7.3%	8565<br>5.4%	31%<br>69%
2	2108<br>1.3%	3028<br>1.9%	7448<br>4.7%	59%<br>41%
	85%<br>15%	72%<br>28%	45%<br>55%	81%<br>19%

Model #7. MCAv PP

Initial

Predicted Class	Target class 0	Target class 1	Target class 2	
0	89612<br>56.0%	19417<br>12.1%	939<br>0.6%	81%<br>19%
1	1750<br>1.1%	31157<br>19.5%	4554<br>2.8%	83%<br>17%
2	26<br>0.0%	7344<br>4.6%	5214<br>3.3%	41%<br>59%
	98%<br>2%	54%<br>46%	49%<br>51%	79%<br>21%

optimal cut-offs

Predicted Class	Target class 0	Target class 1	Target class 2	
0	108581<br>67.5%	1227<br>0.8%	160<br>0.1%	99%<br>1%
1	15461<br>9.7%	12867<br>8.0%	9133<br>5.7%	34%<br>66%
2	1429<br>0.9%	3628<br>2.3%	7527<br>4.7%	60%<br>40%
	87%<br>13%	73%<br>27%	45%<br>55%	81%<br>19%

Model #8. Volumetric

Initial

Predicted Class	Target class 0	Target class 1	Target class 2	
0	103189<br>64.5%	6076<br>3.8%	703<br>0.4%	94%<br>6%
1	2090<br>1.3%	29189<br>18.2%	6182<br>3.9%	78%<br>22%
2	22<br>0.0%	7093<br>4.4%	5469<br>3.4%	43%<br>57%
	98%<br>2%	69%<br>31%	44%<br>56%	86%<br>14%

optimal cut-offs

Predicted Class	Target class 0	Target class 1	Target class 2	
0	108765<br>68.0%	944<br>0.6%	259<br>0.2%	99%<br>1%
1	8880<br>5.5%	17139<br>10.7%	11442<br>7.2%	46%<br>54%
2	361<br>0.2%	3697<br>2.3%	8526<br>5.3%	68%<br>32%
	92%<br>8%	79%<br>21%	42%<br>58%	84%<br>16%

Model #9. HR and BP

Initial

Predicted Class		Target class 0	Target class 1	Target class 2	
	0	89397<br>55.9%	19662<br>12.3%	909<br>0.6%	81%<br>19%
	1	4233<br>2.6%	30542<br>19.1%	2686<br>1.7%	82%<br>18%
	2	173<br>0.1%	8604<br>5.4%	3807<br>2.4%	30%<br>70%
		95%<br>5%	52%<br>48%	51%<br>49%	77%<br>23%

optimal cut-offs

Predicted Class		Target class 0	Target class 1	Target class 2	
	0	108107<br>67.6%	1330<br>0.8%	531<br>0.3%	98%<br>2%
	1	17612<br>11.0%	11305<br>7.1%	8544<br>5.3%	30%<br>70%
	2	1897<br>1.2%	3708<br>2.3%	6979<br>4.4%	55%<br>45%
		85%<br>15%	69%<br>31%	43%<br>57%	79%<br>21%
